# Multiscale Analogue Modelling of Clinching Process to Investigate Thickness Tolerance and Tool Misalignment

**DOI:** 10.3390/ma15103674

**Published:** 2022-05-20

**Authors:** Sia A. Nourani, Dirk J. Pons, Digby Symons, Senlin Zhang

**Affiliations:** Department of Mechanical Engineering, University of Canterbury, Christchurch 8140, New Zealand; sia.nourani@pg.canterbury.ac.nz (S.A.N.); digby.symons@canterbury.ac.nz (D.S.); szh131@uclive.ac.nz (S.Z.)

**Keywords:** clinching, plasticine, multiscale modelling, analogue model, joining quality, thickness tolerance, friction, misalignment, plastic deformation, sheet forming, metal forming, rheology

## Abstract

NEED—The effect of dimensional variability of sheet thickness (tolerance) and tool misalignment is poorly understood for the clinching process. Finite element analysis (FEA) is valuable but requires a lot of and is difficult to verify in this situation due to the asymmetrical geometry and nonlinear plasticity. OBJECTIVE—The objective of this work was to determine the effect of thickness tolerance, tool misalignment and sheet placement (top vs. bottom) in the clinching process, by use of analogue modelling with plasticine. METHOD—Experiments used a scaled-up punch and die, with plasticine as the analogue. Thickness tolerances were represented by sheet thicknesses of 11 and 7 mm, 12 and 8 mm, 8 and 12 mm and 13 and 9 mm for upper and lower sheets, respectively. Two types of lubricant were tested between sheets: glycerine and silicone oil. Angular variability was also introduced. Measured parameters were interlock (also called undercut) and neck thickness. Analogue results for deformation were compared with microscopy of metal clinching. FINDINGS—The results reveal that the multiscale analogue model is an efficient tool for studying the effect of dimensional deviation on a clinch joint. Thickness tolerance showed a critical relationship with interlock, namely a reduction to about half that of the nominal, for both maximum and least material conditions. Increased angular misalignment also reduced the interlock. Compared with glycerine, silicone oil tests showed reduced interlock, possibly the result of a lower coefficient of friction. ORIGINALITY—This work demonstrates the usefulness of analogue modelling for exploring process variability in clinching. The results also show that significant effects for sheet placement are ductility, lubricant (friction), thickness of samples and tool misalignment.

## 1. Introduction

The trends of light-weighting, higher performance and increased functionality are some of the drivers for multi-material, hybrid structures and the need for the joining of dissimilar materials. Furthermore, industries are pressured to use environmentally friendly and energy-saving joining methods. Material joining methods such as welding, riveting, clinching, adhesives and screws are widely in use.

**Figure 1:**
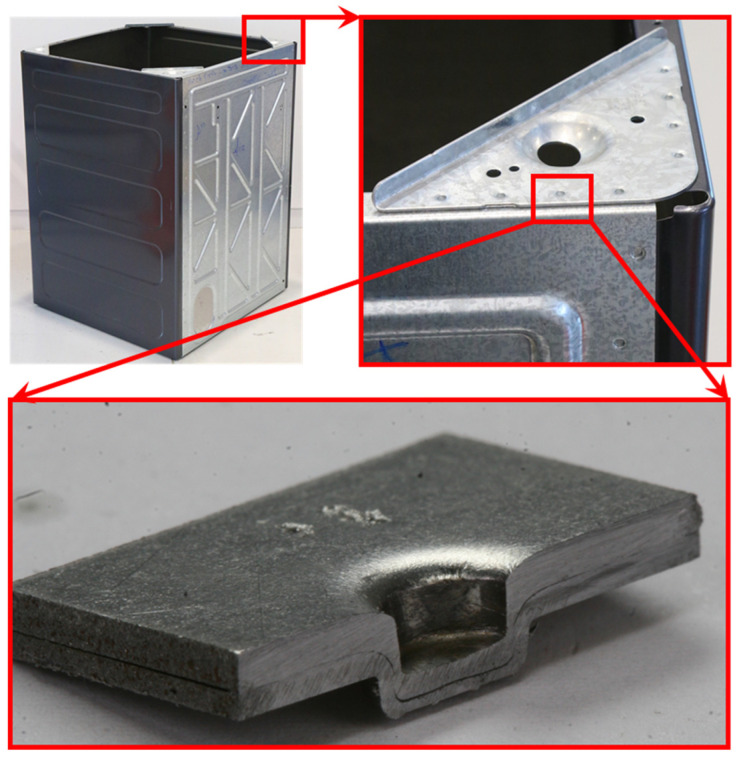
Application of clinching in a washing machine frame.

Clinching is a relatively new high-speed mechanical joining technique suitable for point joining lightweight sheet materials. The only restriction is their plastic properties [1]. Mechanical clinching technology has the ability to connect dissimilar materials, difficult-to-weld materials and coating materials [2,3]. Some areas where clinching is used include the automobile industry, white goods (household appliances), ventilation and air conditioning, electronics, medical appliances and sheet processing in general. Figure 1 depicts the use of clinching in a washing machine frame, where over 100 clinch points are used to create a strong and vibration-resistant frame.

Clinching processes include Die-less Clinching, Flat Clinching, Hole Clinching Technology, Clinch–Adhesive Joint, and others. These processes are still being developed; they require much more research to achieve the point where accuracy, quality, and strength of the joints become comparable to the industry standard [4]. Currently, research on hybrid clinching processes such as ultrasonic assisted clinching, electromagnetically assisted clinching, electrically-assisted mechanical clinching (EAMC), laser assisted clinching, electro-hydraulically assisted clinching and flat-rivet clinching and show that these processes can be adopted on a larger scale [5,6,7,8,9,10].

Since clinching can be used in variety of industries, commercial pioneers in this field (ECKOLD, BTM, TOX and ATTEXOR) have classified their products by tool characteristics. The first and foremost classification is the final shape of the pressed joint. The fundamental distinction between manufacturers using the clinching process is the method to produce joints, which means different die configurations, including expandable and fixed dies [11]. Clinched joints can be either round or square in shape. Round joints have been widely studied because of their greater appeal, despite their inherent limitations when connecting thick and hard plates [12]. Nevertheless, another study showed that a heat-assisted round clinching process can be used to join two limited forming materials [13]. 

There are a number of factors affecting clinch forming, such as interlock between the two sheets, upper and lower sheet thinning, and reduction in the bottom thickness of sheets. Various studies have considered these factors as the main parameters of the process joinability. Inappropriate values of these parameters could eventually lead to one of the following three failure modes in clinched joints: (1) the joint opens because of inappropriate interlocking between the sheets; (2) fracture in the neck area of the upper sheet through low ductility of the material and thickness of sheet materials; (3) a combination of the previous two modes. Lastly, the crack phenomenon in the bottom of the joints is due to the generated tensile stress that can be prevented by eliminating the groove depth i.e., the flat bottom [14,15]. Additionally, these factors are influenced by the tool geometry, mechanical characteristics of the materials, plate thickness configuration, applied forces, and friction [14,16,17,18,19,20].

Many studies have investigated optimization of this process, while neglecting joint quality [21,22,23,24]. However, there are few studies on the effect of deviations and tolerances of parameters on the clinching process. Kam et al. [25] investigated tool eccentricity in clinched joints, and it was concluded that it can influence the quality of joint. The deviation in mechanical and geometrical properties of sheet-metals may negatively affect the interlocking in clinched connections, resulting in poor quality joints. According to Wolter et al. [26] and Tan et al. [27], there are some non-destructive tests and online monitoring systems to control the clinching process in mass production, though the acceptable tolerance in all aspects of sheets is vague. Therefore, studying the effect of deviation can improve interlock accuracy.

The main concern in clinching process is the quality of each single joint or continuous quality control as the clinching process is sensitive to all deviations, particularly in mass production. Varis [28] suggests that there are no unimportant observations and deviations, and that all changes to the clinching process affect the outcome. One of the important factors in the feasibility of joining sheet metals is the material. The mechanical properties of materials used in the joining process play a central role in joint quality. Material formability is determined by mechanical properties such as elongation at fracture, uniform elongation yield stress, tensile strength and hardness. However, these properties typically vary from coil to coil. Hence, monitoring the mechanical properties of the incoming sheet materials is necessary to maintain the quality of joining. In this regard, non-destructive methods such as eddy current testing can be conducted to control the quality of sheet metals [29]. Despite the fact that thickness tolerance of sheets from coil to coil (due to roller deflection) can affect the quality of joints, the impact of this deviation has never been studied until now.

Misalignment can cause tool damage and reduced joint strength. The joints must be formed perpendicular to the metal surfaces. All of the clinching tool sets within the die must be set up to bottom simultaneously to produce consistent button dimensions for all of the joints. The maximum misalignment of a clinching tool was reported to be three degrees [28]. This process parameter has also never been investigated and the impact of misalignment on quality of joint is poorly understood, while it is argued by Abdul Ghafar, et al. [30] that the impact of misalignment on tool life is highly important. 

Finite element analysis (FEA) is valuable to analyse the clinching problem, but effortful and difficult to verify in this situation due to the asymmetrical geometry and nonlinear plasticity. Examples of FEA clinch models are [31,32,33,34]. Hence, there is value in considering other methods that can elucidate the flow of material in the plastic deformation, especially methods that are quick to deploy. Plasticine analogue is a simple example for representing metal forming processes in general, as well as for indicating detailed patterns of plastic deformation behaviour [35,36,37]. This is particularly a need in the case of asymmetric geometric arrangements, since assumptions of symmetry are much reduced, hence making for difficult FEA implementation. Analogue modelling with plasticine has the potential to alleviate this problem.

Aluminum and other metal sheets in the clinching process require high stress from the punch, and it is also difficult to control the thickness tolerances. However, these factors can be avoided by using plasticine layers. The plasticine layers require less stress from the punch, and it is easy to control the tolerance in an ideal range. Plasticine can be colored in a variety of ways to increase the experiment’s visibility. Plasticine, like metals, exhibits stress–strain behavior at both high strain rates and temperatures. Applications of plasticine in analogue modelling include stamping, rolling, forging, Friction stir welding (FSW) and rock structure [38,39,40,41]. To the best of our knowledge, there is no report in the literature on the clinching process of plasticine analogue modeling, and only a few papers have been published on the metal forming.

**Figure 2:**
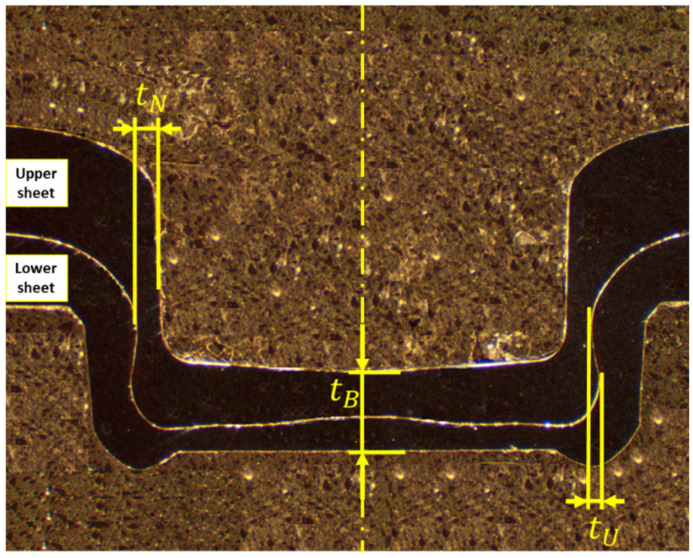
Cross section of clinch joint: neck thickness $\left(t_{N}\right)$, undercut/interlock $\left(t_{U}\right)$ and bottom thickness $\left(t_{B}\right)$.

In this study, a plasticine analogue model was used as a cost-effective alternative. The aim was to straightforwardly accommodate variability in geometric tolerances. The tolerances of interest are thickness and misalignment. Therefore, the feasibility of a plasticine analogue model was investigated to ensure a qualitative model could be produced. Following this, the effect of different configurations of sheet thickness tolerances and tool misalignment on the main quality parameters of clinching, such as interlock (also called undercut) $\left(t_{U}\right)$, neck thickness $\left(t_{N}\right)$ and bottom thickness $\left(t_{B}\right)$ were explored (Figure 2). 

## 2. Methodology

### 2.1. Approach

This work had several objectives. The first was to evaluate the representativeness of plasticine as an analogue model for steel clinching, and to find a way to produce repeatable results. The second was to determine the effect of thickness tolerance, tool misalignment and sheet placement (top vs. bottom) in the clinching process. A third objective was to provide a qualitative understanding of the plastic flow features. 

The overall approach (described in more detail below) was to conduct experiments with both a conventional steel tool and steel sheets, and a 10:1 scaled-up punch and die with plasticine. To compare the qualitative evolution of the force profile during clinching, the punch force was measured in both cases, for representative samples. Thickness tolerances were represented by plasticine sheet thicknesses of 11 and 7 mm, 12 and 8 mm, 8 and 12 mm and 13 and 9 mm for upper sheet and lower sheet, respectively. Two types of lubricants were considered between sheets: glycerine and silicone oil. Angular variability was also introduced. Measured parameters were interlock (undercut) and neck thickness. Analogue results for deformation were compared with microscopy of metal clinching.

### 2.2. Clinch Forming

Firstly, an actual round TOX clinching process for sheet-metal was conducted along with a plastic deformation. The main geometric parameters of the joints, such as neck thickness $\left(t_{N}\right)$, undercut $\left(t_{U}\right)$ and bottom thickness $\left(t_{B}\right)$, were measured using an optical microscope. Based these results, a scaled up analogue model of the clinching process was established. The qualitative analogue model was compared and calibrated with the actual clinching process. Finally, the analogue model was used to explore the effect of different configurations of sheet metal thickness tolerances and tool misalignment on the quality parameters of clinching.

### 2.3. Actual Clinching Process

The most important part of this analogue modelling study was to ensure that the plasticine analogue model could reproduce similar results to the real process. Therefore, experimental tests were conducted to compare and establish the plasticine simulation of the clinching process.

**Figure 3:**
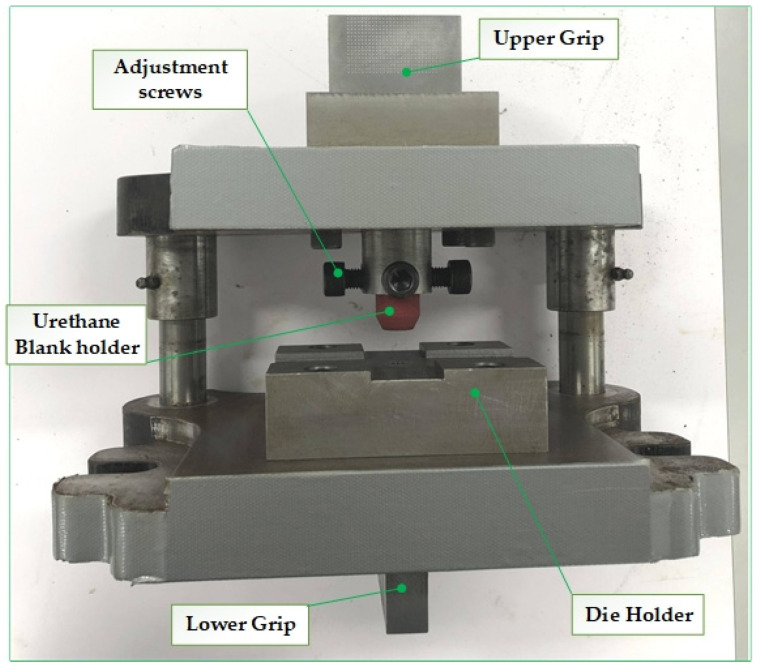
Experimental setup: Clinching die set.

The TOX clinching system consists of a fixed die, a punch, a blank holder and a stripper. In this system, as the punch moves down, the blank holder has to gradually exert pressure on the upper sheet. Adequate blank holder force and contact surfaces are essential to achieve a defect free connection. A die set was designed in such a way to be mounted in a computer-controlled servo-hydraulic testing machine (MTS^®^ 810 Material Testing System). This setup (Figure 3) allowed for easy control of the press stroke and force measurement.

Commercial cold rolled steel sheet (JIS G3141-SPCC SD) was used with thicknesses of 1.2 mm and 0.8 mm for upper and lower sheets, respectively. Sheets of the same thickness were cut from one coil to ensure equivalent mechanical properties. The variation of the thickness over the width of a SPCC-coil has been reported to range from 50 μm to 70 μm. Chemical and mechanical properties of SPCC material were reported by Nourani et al. [42]. The servo-hydraulic testing machine applied a compressive force and measured the displacement of the punch. Total movement of the punch was 2.9 mm and the processing time approximately 1 s.

The punch and die are commercially available in the market, thus the shape and dimensions are determined and standardized by TOX^®^ PRESSOTECHNIK. Clinched specimens of 6 mm diameter were made by a TOX^®^ clinching die set consisting of a punch (TOX part # 10.180), a round fixed die (TOX part # 10.25) and an elastic blank holder (red color Lurethane^®^ with durometer hardness value of 90 A in accordance with the standard ASTM D575-91). A total of five optical microscopy specimens were created using the same process parameters. In all cases, the geometric and process parameters resulted in a robust clinch joint.

Joints were cross-sectioned with a low-speed saw (ISOMET^®^). Specimens were mounted in resin and polished using colloidal silica to 0.02 μm (Buehler^®^ Beta 2 dual platen grinder–polisher machine equipped with Vector power head). The main geometric parameters of the joint were measured using an optical microscope (Olympus SZH10, Auckland, New Zealand). Next, specimens were etched with 2% Nital solution for imaging via optical microscopy [42].

### 2.4. Analogue Modelling with Plasticine

The approach in this survey was experimental rather than theoretical, and involved:

1. A scaled-up punch and die set were designed and constructed, nominally 10:1. Aluminum was used to make the punch and die, while hardened steel was used to make the die set. Die-set guides were designed to function as stroke end blocks (for determining the bottom dead center of the die), ensuring that the distance between the punch and die after clinching was exactly 7 mm. To prevent air entrapment, a hole of 4 mm was drilled in the die. The blank holder was eliminated from this design because the plasticine was too soft and the material would be entirely warped and squished if it was used. Eventually, various techniques were used to assess the accuracy of the die set in the workshop and the geometrical tolerances of the punch and die were found to be less than ±0.05 mm (Figure 4).

**Figure 4:**
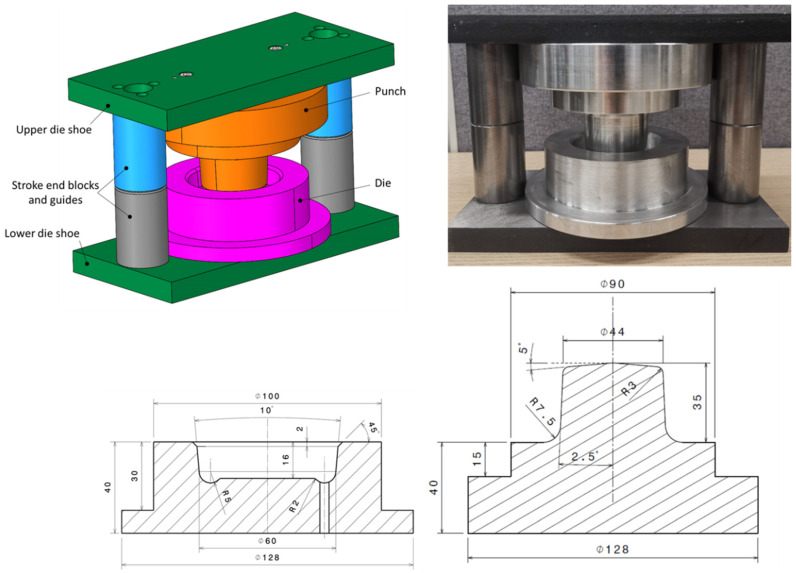
Scaled-up die set for plasticine analogue model of the clinching process (dimensions in mm).
2. Multi-layered plasticine slabs were fabricated under required sizes, representing sheets in the clinching process. Different colors of plasticine manufactured by New Clay Products United Kingdom were employed. Manually, each color of plasticine was rolled to a consistent thickness of 1 mm using a rolling pin and an aluminum stencil frame with dimensions of 100 mm × 100 mm × 1 mm (length × width × depth), see Figure 5. Several lubricants were tested for rolling each plasticine including water, silicon oil and glycerin (with kinematic viscosities of 1, 100 and 1100 cSt, respectively) to prevent the plasticine from attaching to the roller and aluminum stencil frame. Results indicated that glycerin was the superior option. Layers of different colors of plasticine were stacked on top of each other. To improve the adhesion between the layers, the stack was rolled again with a small amount of pressure. The latter of these stacked up layers was trimmed to the required dimensions using a thin cutter of 0.5 mm thickness. A small amount of glycerin was applied to the edges of the cutter to avoid mixing of different layers while cutting. The thickness tolerance of the manufactured slabs was kept within ±0.1 mm.

**Figure 5:**
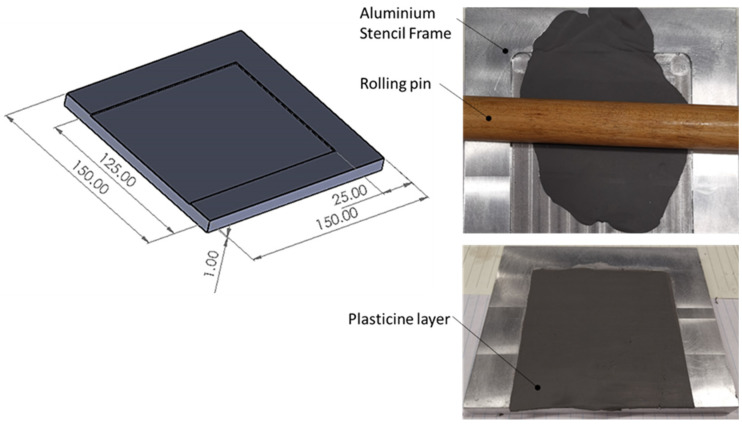
Tools to make plasticine layers of 1 mm.
3. A manual press was used to complete the clinching process for a total of 34 samples of plasticine (Figure 6). In the first stage, 16 experiments were carried out to investigate the feasibility of a plasticine analogue model in the clinching process, with upper and lower sheets measuring 12 mm and 8 mm, respectively. To achieve this, the top and bottom of the slabs were lubricated thoroughly (or else the plasticine would stick to the punch and die) before being placed on the die in the desired combination. When the handle is pressed, the rack is pushed downward. Thus, the punch compresses the plasticine sheets down into the die to the bottom dead center, completing the clinching process. Once a reliable plasticine analogue model was established, a force–displacement diagram was obtained through linear regression from three samples. The applied force in the clinching process was accurately measured using a S-type load cell (YZC-516C (200 kg)). The load cell was connected to a cDAQ-9174—NI—National Instruments module to measure the compressive force in the deformation process of plasticine.
4. To prevent sample damage, the die-set was placed in a freezer for two hours to ensure that the clinched plasticine was hard enough to be removed from the die-set. Plasticine layers were cut into two sections using stainless steel wire (SS 316L) with a diameter of 0.2 mm.

**Figure 6:**
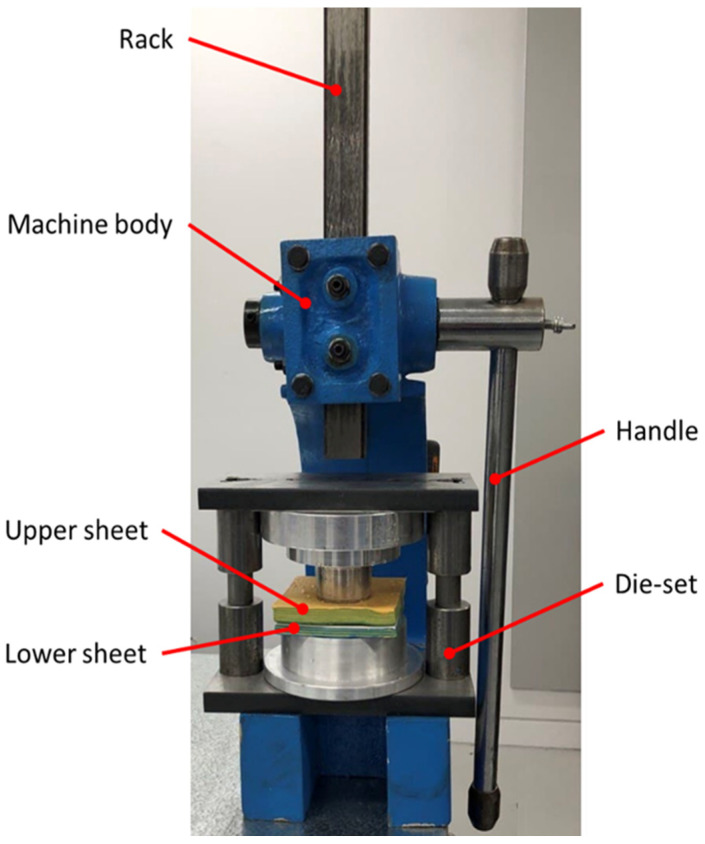
Scaled-up clinching die-set set up mounted in a manual press.
5. A digital camera (Canon EOS 30D DS126131 8.2 MP with Canon Ultrasonic Zoom Lens) was used to photograph cross-sections. Subsequently, an optical microscope (OLYMPUS SZH10, Auckland, New Zealand) was used to measure the bottom thickness, interlock and neck thickness accurately. The first step to measure interlock and neck thickness was to find the interface between upper sheet and lower sheet. Samples were then compared with the cross-section of an actual steel clinched joint.
6. Different configurations of the main experiments are summarized in Table 1. The first four sets of experiments (S1 to S4) were conducted to assess the reliability of the plasticine analogue model. Next, the thickness tolerance of upper and lower sheets was explored. Variation in thickness tolerance was applied only when the upper sheet was the thicker one (experiments S4, S5 and S6). Experiments were conducted only for a critical range of thickness tolerances. Since the results of each set of experiments were close enough, all the experiments were carried out in duplicate for each configuration, and mean values are reported (unless the results were unexpected, in which case additional experiments were conducted.)

Finally, two sets of experiments (S7 and S8) were conducted to determine the effect of angular misalignment on clinched joints. Cold-rolled steel strips were shimmed between the punch and upper die shoe to produce an angular misalignment. Inserting a 1.5 mm shim on one side of the punch results in a 1.5° misalignment. It was not possible to tilt the punch more than 1.5° as the dowel pins did not allow this. Therefore, two steel spacers with thicknesses of 1 mm and 0.5 mm were used to angularly misalign the punch 1° and 1.5°, respectively (two tests for each set were replicated to study the misalignment using the plasticine analogue model).

## 3. Results and Discussion

### 3.1. Establishing a Method for Plasticine Clinching

#### 3.1.1. Preliminary Results and Production Issues

**Figure 7:**
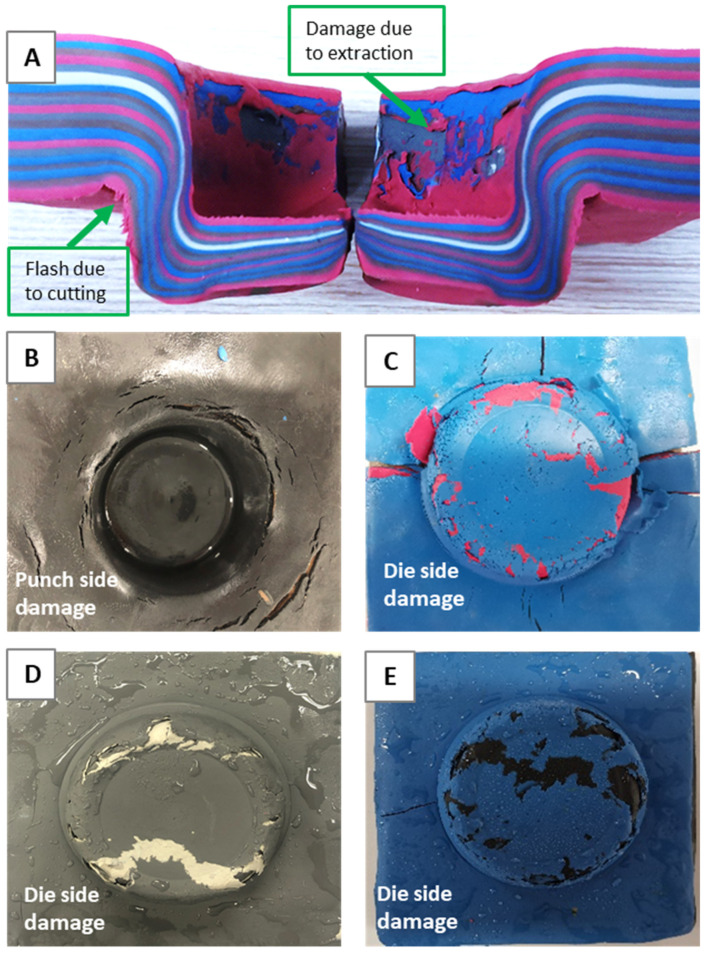
Preliminary results for plasticine clinched joints: (**A**) damage due to extraction and flash due to cutting; (**B**,**C**) damage caused by the use of silicon oil to lubricate the upper and lower sheets; (**D**) damage due to excessively cooling the sample; (**E**) damage due to preheating the slabs.

It was necessary to fine-tune the plasticine technique and overcome key difficulties with the analogue modelling. Sixteen clinched samples of 12–8 mm (combination of upper sheet 12 mm and lower sheet 8 mm thick) were tested to establish a reliable analogue model. Damage and tears in the plasticine samples during the process were studied to improve the analogue modelling technique and find the appropriate clinching parameters including lubricant, temperature and process time. The preliminary results of the plasticine clinching process, which are of considerable importance to reproduce a damage free plasticine joint, are depicted in Figure 7 and summarized as below:

**A:** Damage due to extraction and flash due to cutting—Process time 1 s.

The flash occurred after using the cutter to cross section the plasticine samples. The results revealed that, if the specimen was not chilled after clinching, extraction damage occurred on both die side and punch side. The key reason is that plasticine at room temperature is too soft and could easily be damaged during the removal of the punch and die. To address this issue, a series of tests were carried out in which plasticine-clinched samples were frozen at time intervals ranging from 0 to 5 h, with the best outcome obtained with a 2-h freezing period following clinching. Another factor is friction between tools and plasticine slabs, which could be improved by lubricating the slabs.

**B&C:** Damage caused by the use of silicon oil to lubricate the upper and lower sheets—Process time 5 s.

Different lubricants such as water, glycerin, and silicon oil were tested, and the findings indicated that, while silicon oil with a lower viscosity effectively decreased friction, the chemical interaction between plasticine and silicon oil could soften the plasticine, resulting in sample tearing and damage. Water as a lubricant was also studied, but the results were inadequate in the initial modelling attempts; therefore, it was excluded from the list. This was consistent with findings from other investigations [40].

**D:** Damage due to excessive cooling of the sample on die side—Process time 5 s.

In other tests, the upper and lower slabs were cooled to −4 °C before the clinching process. These samples were placed in the freezer for 1 to 4 h. Damage was attributed to embrittlement of the plasticine. It was presumed that the damage occurred during the early stages of the clinching process, when the material was in tension. This method could be beneficial to compare the analogue model with the actual clinching process in hard material sheets. However, in this study, the focus was on ambient temperature and ductile materials.

**E:** Damage due to preheating the slabs before clinching—Process time 5 s.

Preheating the slabs before clinching was conducted using an oven to evaporate glycerin. The process included heating the oven up to 60 °C in half an hour, putting the plasticine slabs inside the oven for two hours and then cooling down the samples in the room temperature. However, the material became brittle and it was easily damaged.

#### 3.1.2. Improvements to Technique

After conducting the above tests to search for a reliable analogue model, the improvements below were used to produce consistent results without damage for further experiments:

- The plasticine clinching process should be performed at room temperature and any freezing or preheating the slab before clinching is not recommended in this application.
- Using glycerin instead of silicon oil and water as the results were more reliable.
- The clinching process was timed from 1 to 10 s, and the results indicated that 1 s was surprisingly the best process time to avoid damage.
- Clinched samples were kept in the freezer for two hours with plastic bag and desiccant which could absorb the moisture in the freezer.

### 3.2. Sensitivity and Calibration

The shear stress between the plasticine layers was one of the key challenges in this investigation. When compared to genuine sheet metal clinched joints, it was essential to ensure that the friction between the layers was sufficient to create a reliable analogue model that accurately simulated an actual sheet metal clinched joint. Additionally, in order to obtain consistent results, the base plasticine model must be calibrated. This was accomplished through the conduct of four sets of experiments and discussion of the results.

#### 3.2.1. Comparison of Interlock Combinations 12–8 vs. 8–12

**Figure 8:**
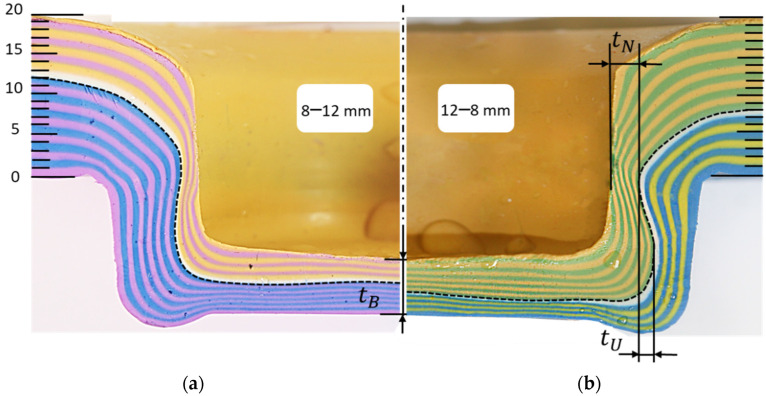
Comparing the final shape of experiments: (**a**) left; inverted combination (S1: 8–12 mm) and (**b**) right; base model (S4: 12–8 mm) in single stroke clinching of plasticine.

Figure 8 illustrates the cross-section with interlock $t_{U}$ and neck thickness $t_{N}$ which compares the final geometry of the base plasticine clinched joint (right) and the inversed configuration of upper and lower sheets (left) in this study. The number of each layer was identified, and the interlocks were measured across all the layers in both samples, see Table 2. For experiment 12–8, twenty layers of 1 mm thick plasticine were used: 12 layers as the upper sheet and 8 layers as the lower sheet. The glycerin was used to lubricate the top and bottom sheets, and punch and die. The clinching process was operated in 1 s and the samples were stored in a freezer, in a plastic bag with desiccant, for two hours after clinching. In another experiment, the combination of upper and lower sheet was inverted with otherwise the same conditions of testing, 12 layers as the lower sheet and 8 layers as the upper sheet. Layer 1 exhibited no interlocking and no appreciable damage in all measured samples, despite being thinned in the basal plate. Although layer 20 was thinned to the point of failure in necking regions, it lacked interlock as well.

Results of neck thickness $\left(t_{N}\right)$ and undercut $\left(t_{U}\right)$ for both sets of experiments were 3.11 and 1.68 mm for the base plasticine joint, respectively, and 1.91 and 0.84 mm for the inverted configuration sample. The observed decrease in neck thickness and undercut suggest that a link may exist between the order of upper sheet and lower sheet and mechanical behavior of plasticine clinched joints as the thicker sheet should be on the punch side to produce a stronger joint. The present findings seem to be consistent with other research, which found that, when the thicker material is located on the punch side, the potential for a round joint shape is greatest [43]. Furthermore, inverting the configuration of upper and lower sheets in the plasticine analogue model results in a considerable change in the maximum shearing force value, which may cause failure during the joining process.

**Figure 9:**
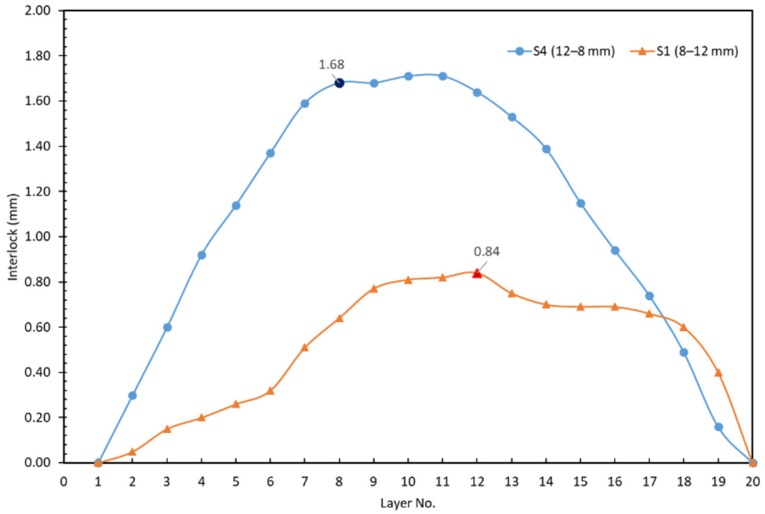
Comparison of interlock for experiments S4 (12–8 mm) and S1 (8–12 mm). The numbers refer to the thickness of the top and bottom sheets, respectively. The emphasized markers show the interfaces. Layers are numbered from the bottom.

Figure 9 shows the interlock for each layer between experiments S4 (12–8 mm) and S1 (8–12 mm). The interlock of the 12–8 mm sample is approximately two times better than that of the 8–12 mm sample. The differences between the 12–8 and 8–12 samples show that the samples were not behaving as solid blocks, i.e., there was an element of shear occurring on the interface layer.

#### 3.2.2. Joint 12–8 with Simple Two-Slab Layup

**Figure 10:**
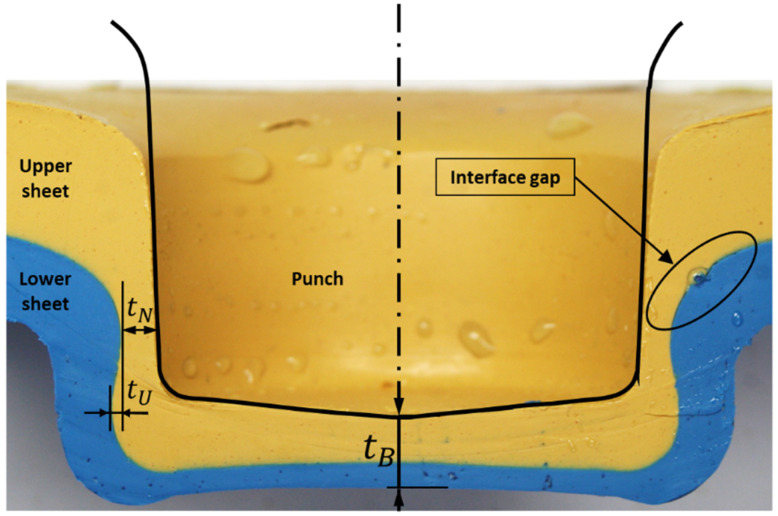
Cross section of plasticine clinch joint with two slabs.

Following this, a set of experiments (S2) was conducted with two slabs that were cross sectioned in order to compare the results of the neck thickness $\left(t_{N}\right)$ and undercut $\left(t_{U}\right)$ with the base plasticine sample (S4). The recorded values were 3.14 and 1.14 mm respectively, see Figure 10 and Figure 13. The test was successful as it was able to produce similar results for neck thickness in comparison to the base plasticine sample, even though the undercut was approximately 30 percent smaller. This discrepancy in undercut is attributed to higher shear strength in the monolithic slabs compared to the composite slabs with their many internally (potentially partially lubricated or softened) layers.

The main purpose of this set of experiments was to study the sensitivity of the plasticine multi-scaled model to number of layers for each slab in the clinching process. However, using only two slabs of plasticine instead of multiple layers could be faster and easier, especially in studying the thickness tolerance and tool misalignment. Further work is required to establish this.

#### 3.2.3. The Role of Lubricant: Comparison of Interlock Combinations 12–8 mm (Glycerine vs. Silicone Oil)

**Figure 11:**
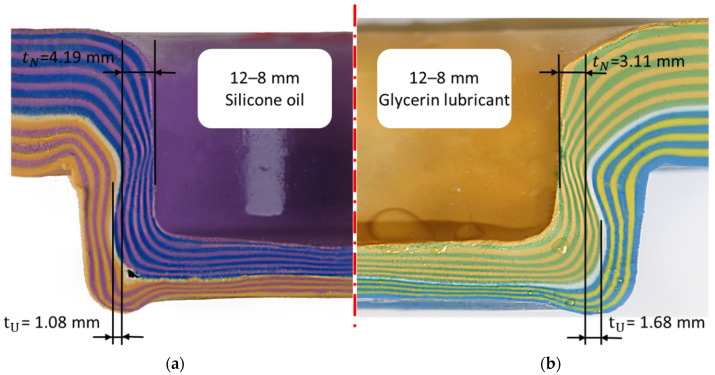
Comparison between cross section of plasticine clinch joint using glycerin and silicone oil: (**a**) left; silicone oil lubricant (S3) and (**b**) right; glycerin lubricant (S4).

Figure 11 shows the cross-section with interlock $t_{U}$ and neck thickness $t_{N}$ which compares the final geometry of the experiment S4 with glycerine lubricant (right) and the experiment S3 with silicone oil (left). In experiment S3, similar to the previous set of experiments, the interlocks were measured across all layers, see Table 3. Layer 1 lacked interlock and sustained little damage, despite being thinned in the basal plate. The layer 20 had no interlock, but this layer was thinned, potentially to the point of failure at necking regions.

**Figure 12:**
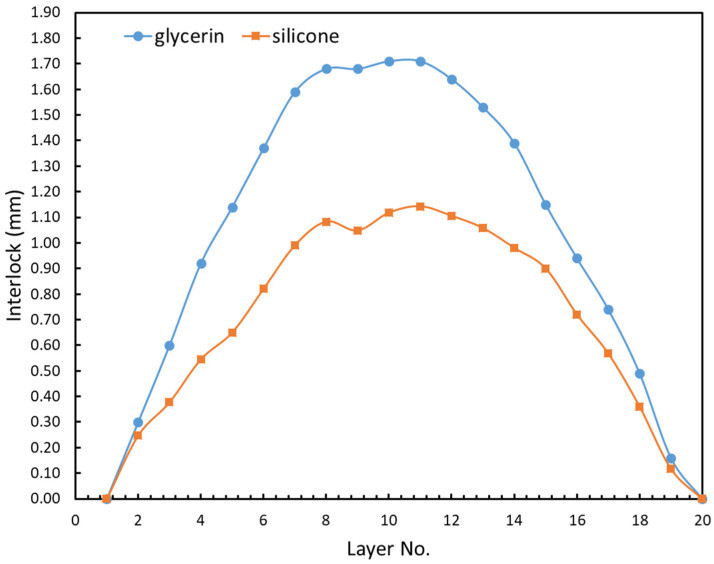
Comparison of interlock between experiments S3 and S4 (12–8 mm, silicone oil and glycerin).

Lubrication refers to the type of lubricant used and its effect on the interlock. Lubricant serves many purposes, but the ultimate goal of a lubricant is to reduce unwanted friction (the resistance encountered when solid surfaces slide against each other). This friction reduction was accomplished by separating two solid surfaces with a thin layer of lubricant. Adding lubricant between the plasticine slabs, punch and die could avoid the damage during extraction of the sample. Figure 12 compares the interlock between experiments S4 (12–8 mm, glycerin) and S3 (12–8 mm, silicone oil). The interlock of the glycerin sample was approximately two times better than that of the silicone oil sample, and it also provided a better representation of the clinching of steel. Additionally, it is true that the interlock between layers steadily increased up to the interface and then decreased to zero for layer 20 as a result of the slabs sliding against one another.

**Figure 13:**
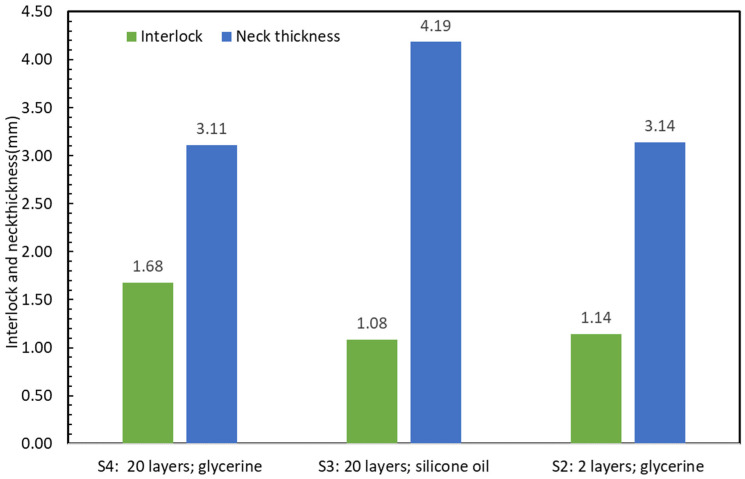
Comparison of interlock and neck thickness between experiments S2, S3 and S4.

Figure 13 compares the interlock and neck thickness between three sets of experiments including S2 (12–8 joint, only 2 layers of plasticine with glycerin lubricant), S3 (12–8 joint includes 20 plasticine layers with silicone lubricant) and S4 (12–8 joint, base plasticine model with 20 layers of plasticine with glycerin lubricant). The results of these two different lubricants revealed a significant decrease in interlock for the silicone lubricant and a significant increase in neck thickness (compared to glycerin).

The coefficient of friction for silicone is believed to be about 0.15 [44] and for glycerin 0.383 [45]. The above results suggest that the lower coefficient of friction of silicone may be the cause of the reduced interlock. The present findings seem to be consistent with other research which found that increasing the friction factor between the joined sheets increases the interlock value, but reduces the neck thickness [46]. A possible explanation is that the formation of the interlock relies on shear coupling between the two sheets, and, if this is reduced by lubrication, then the sheets are able to slip against each other at the interface and deform independently of each other.

Tentatively, this implies that, for clinching in steel, it might be possible that oily surfaces might result in less interlock, hence poorer quality joints. This also raises questions about the role of surface coatings more generally, such as electragol and preprint, as these may be softer in shear than the steel substrates.

### 3.3. Validation of the Equivalence of Plasticine Model vs. Actual Clinch Joint

The focus of this section is to compare the overall geometric equivalence and microstructure evolution of the base plasticine model and actual clinch joint.

**Figure 14:**
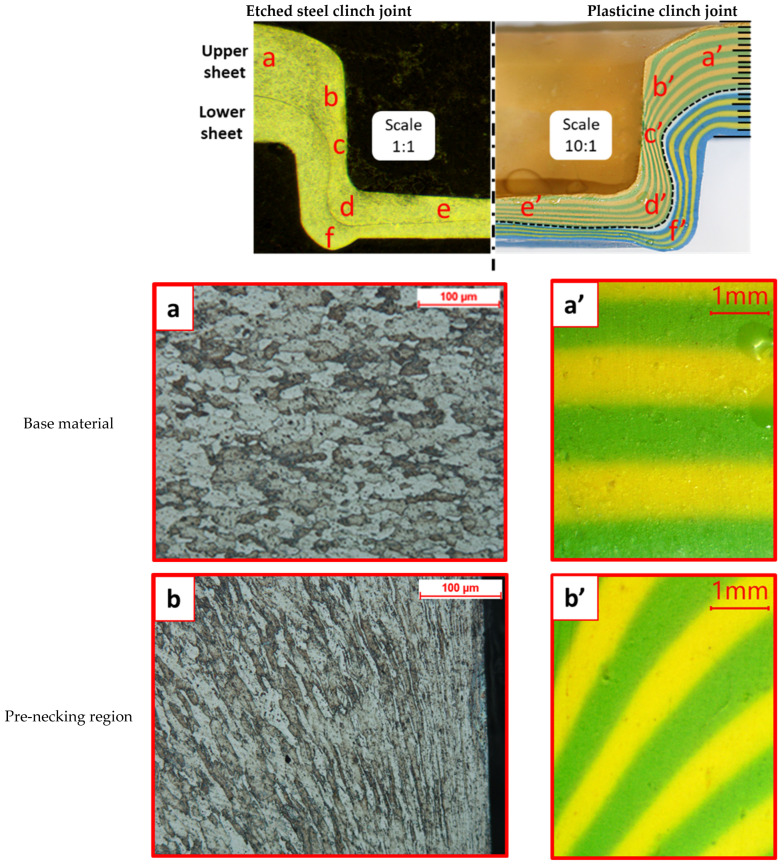

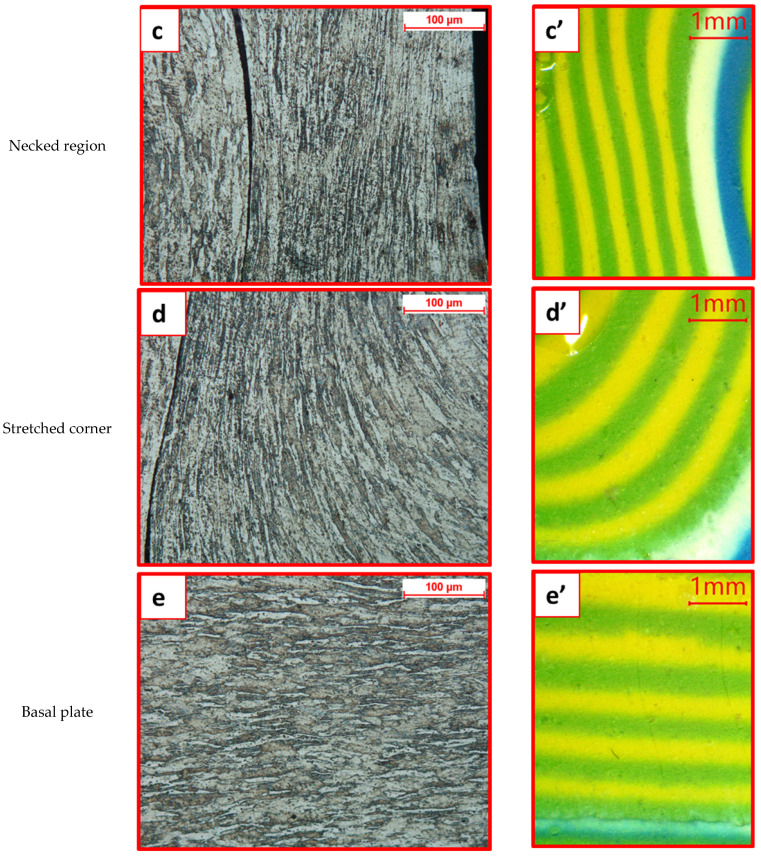

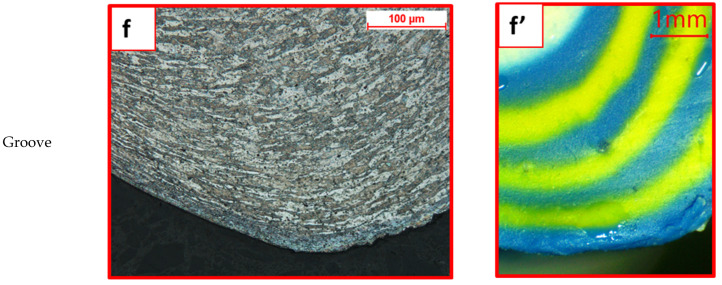
Cross sections of steel and model plasticine clinched joints: (**a**,**a’**) base material, (**b**,**b’**) pre-necking region, (**c**,**c’**) necked region, (**d**,**d’**) stretched corner, (**e**,**e’**) base plate and (**f**,**f’**) groove.

The cross sections of a 12–8 plasticine joint (base model) were compared to a clinched joint made from SPCC mild steel: sheet thicknesses 1.2 mm and 0.8 mm, identical tool geometry to 10:1 scale, sectioned polished and etched (see method). Observations were made with an optical microscope. The main regions in the cross section of clinched joint were examined: base material, pre-necking region, necked region, stretched corner and basal plate. Results are illustrated in Figure 14.

Inspection reveals that the plasticine deformed sample had the same structural anatomy as SPCC mild steel in terms of orientation of colour bands and grains, respectively. The results also had a good agreement with other microstructural studies of steel clinched joints [42,47,48]. It is concluded that there is a correspondence at the microstructural level between the plasticine analogue model and the steel joints.

In addition, the main quality parameters of the mild steel and plasticine joints were measured. The average values of the neck thickness $\left(t_{N}\right)$ and undercut $\left(t_{U}\right)$ were 0.30 and 0.13 mm for the steel clinch joint, and for plasticine (×10 scale) 3.1 mm and 1.68 mm, respectively. The geometric similarity suggests that the clinching process has an element of material conservation at work.

#### Force Diagram Analysis

A significant force is required to complete the clinching process in sheet metals. Elastic deformation and deflection (which is typically restricted to a value of 0.6 mm.) occurs in the clinching machine structure due to the increased force [28]. As a result, the machine’s deformed structure returns to its original position once the punch moves upward after the clinching process is complete. It is therefore necessary to consider the machine’s stiffness, which acts like a spring constant, when calculating the total applied force during the process of clinching. As a result, the force–displacement diagram for the clinching process should be modified to account for actual force (punch force).

**Figure 15:**
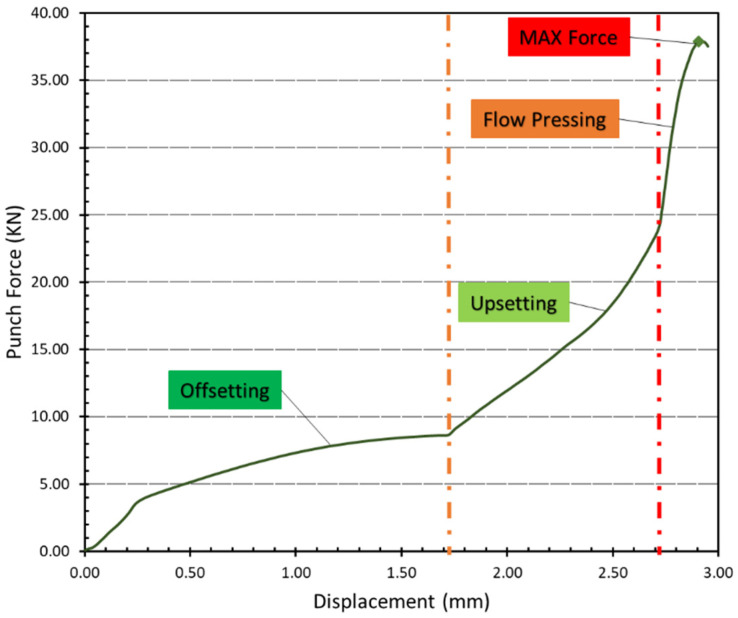
Force–displacement diagram of clinch joint SPCC mild steel.

The modified form of the punch force and displacement of the SPCC sheet metal clinched joint was determined per Coppieters et al. [15], and results are shown in Figure 15. In this particular clinch design, prior to offsetting, a force is required to hold the sheets to the die, which is applied by the blank holder. In the “offsetting” step, plasticizing the material requires a significant increase in force [49]. Between the punch and the bottom of the die, the increasing material upsetting causes the material to flow radially, forming the undercut between the two pieces of sheet metal to be connected. At the point of the maximum punch stroke (2.90 mm), the maximum force (37.84 KN) is reached.

**Figure 16:**
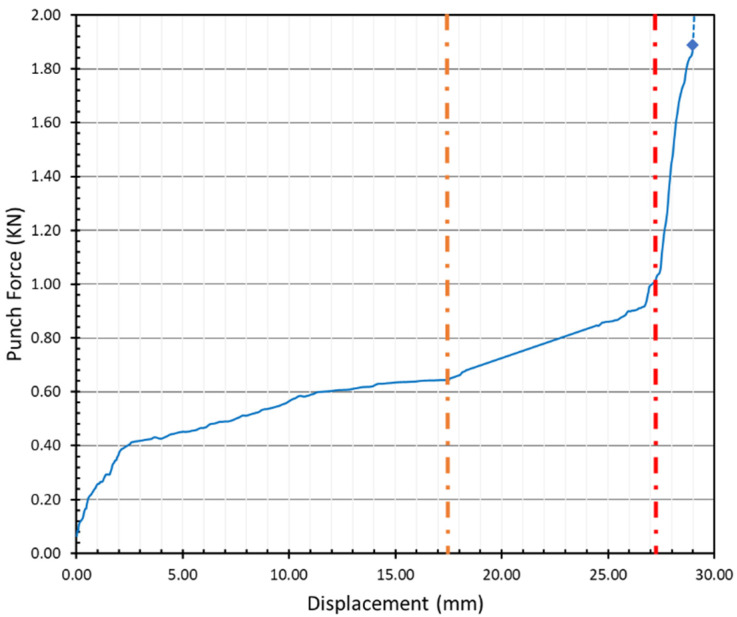
Force–displacement diagram of base plasticine clinch joint (12–8 mm) with glycerin and process time of 1 s.

Even though the blank holder was not included in the plasticine analogue model, plasticine clinch joints followed a similar pattern to steel clinch joints, see Figure 16. The maximum forming force in this application depends on the materials, temperature and the frictional condition, but the average measurement results indicate that the maximum applied force in the plasticine clinching process was roughly 1.89 KN.

Force is an indication of the rheology, how the sheet material flows into the mould volume provided by the punch and die. This is a complex process because the early stages involve elastic deformation in bending of the sheets (i.e., the loading is transverse to the material plane), followed by the development of in-plane tensile deformation that culminates in a necking process. Towards the end of the process, there is bulk compression of the material and lateral flow to fill the groove and conform to the cavity created by the tooling. In addition, the circular nature of the geometry adds circumferential strain effects. The strain regime changes throughout the process, as evident in the changes (some abrupt) in the stiffness profiles of the above figures. The rheology represented in the final joint is a consequence of complex interactions between these various temporally changing strain fields.

### 3.4. Thickness Tolerance: Joints 11–7 mm and 13–9 mm

**Figure 17:**
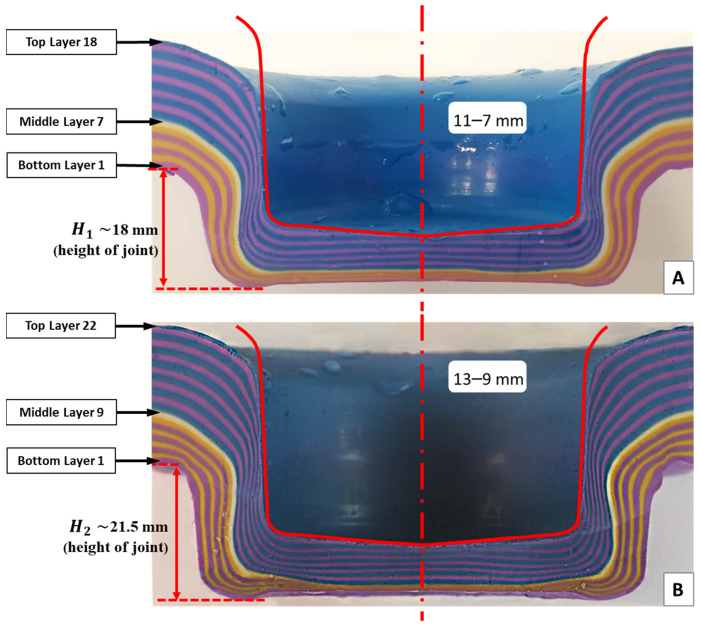
Cross section of plasticine clinch joint experiments: (**A**) S5 plasticine joint 11–7 mm; (**B**) S6 plasticine joint 13–9 mm.

Two sets of experiments (experiments S5 and S6) were conducted to investigate the effect of thickness tolerance in clinched joints by adding and removing the layers on both upper sheet and lower sheet. Figure 17 shows the cross section of experiments S5 (11–7 mm) and S6 (13–9 mm). The interlocks were measured across all the layers in both experiments, see Table 4. Results of neck thickness $\left(t_{N}\right)$ and undercut $\left(t_{U}\right)$ for both sets of experiments were 2.79 and 0.62 mm for the joint 11–7 mm, respectively, and 3.42 and 0.83 mm for joint 13–9 mm.

#### Sheet Tolerance: Optimal Stacking of Sheets of Different Thickness

**Figure 18:**
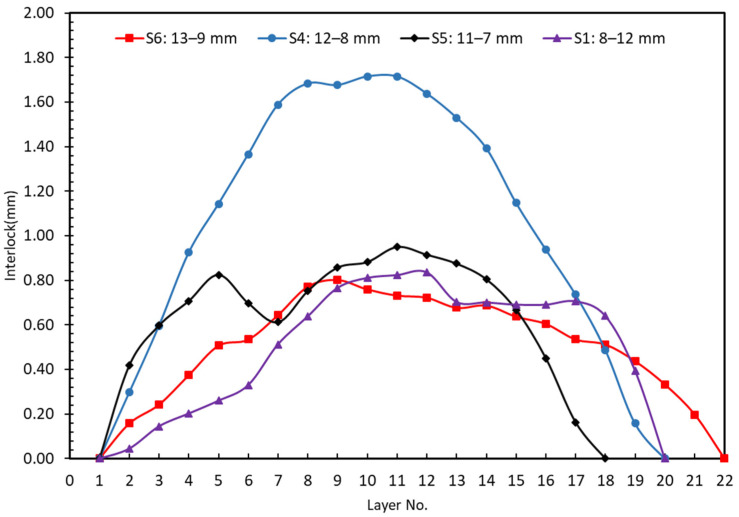
Comparison of layers interlock between S1, S4, S5 and S6.

There are three types of material conditions considered: the nominal material condition is the 12–8 joint sample; the maximum material condition (MMC) is the 13–9 joint sample, and the least material condition (LMC) is the 11–7 joint sample. Figure 18 shows that the interlock of the 12–8 joint sample is the largest among these four plasticine samples and the value is 1.68 mm. Therefore, the optimal stacking of sheets is 12–8 sample (12 mm as upper sheet and 8 mm as lower sheet). Additionally, the results show that the quality of the clinch joint is sensitive to thickness tolerance, with a large reduction in interlock (down to approximately 40% of nominal material condition value) for 9% maximum and minimum tolerance variation. Tentatively, this implies that, for clinching in steel, assuming a 10:1 scale, a sheet thickness tolerance of ±0.10 mm could have a significant reduction in interlock for the MMC and LMC. The mean tolerance on commercial sheet is typically 0.10 mm [50].

**Figure 19:**
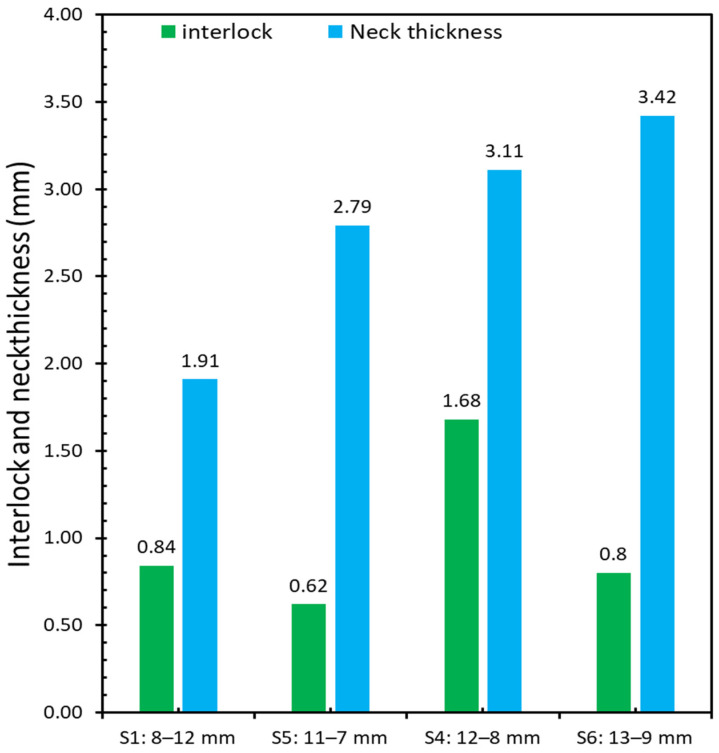
Comparison of interlock and neck thickness between S1, S4, S5 and S6.

In Figure 19, the results of experiments S1, S4, S5 and S6 with various combinations of sheet thickness were compared. What can be understood from this figure is that the variation in thickness tolerance between different combinations of upper and bottom blanks leads to significant changes in values of interlock. While experiment S6 (13–9 mm) had the thickest neck, increasing the thickness tolerance of both the upper and lower sheet results in a significant decrease in interlock. This decrease was unexpected, but it could be explained by conservation of mass. Once the die was filled out and chattering or counter-piping happened (too much material into a die causes counter-piping), plasticine moves out of the die. This is also evident in Figure 17, where the height of the joint in experiment S5 (11–7 mm) is less than experiment S6. Additionally, in experiment S5, the combination of upper and lower sheets is the most critical condition, resulting in a −59.60% decrease in interlock value. These findings support the idea that experiment S4 is the optimal combination.

### 3.5. Angular Misalignment Experiments: Joint 12–8 and 1.5 Degree of Punch Misalignment

**Figure 20:**
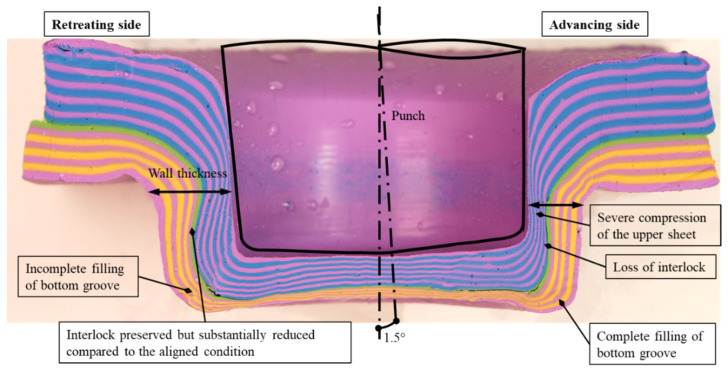
Description figure of 1.5° of punch misalignment (12–8 mm) with glycerin lubricant.

The study’s final step involved angularly misaligning the punch. Figure 20 shows the cross section of overall geometry for experiment S8. In this set of experiments, the punch was misaligned by inserting a shim of 1.5 mm. The interlocks and neck thickness were measured and reported as 1.30 mm and 4.34 mm for the retreating side and 0.34 mm and 2.66 mm for the advancing side. The experiment S7 under 1 degree punch misalignment was conducted as well. The interlocks and neck thickness were measured and reported as 1.41 mm and 3.72 mm for the retreating side and 0.52 mm and 2.90 mm for the advancing side.

**Figure 21:**
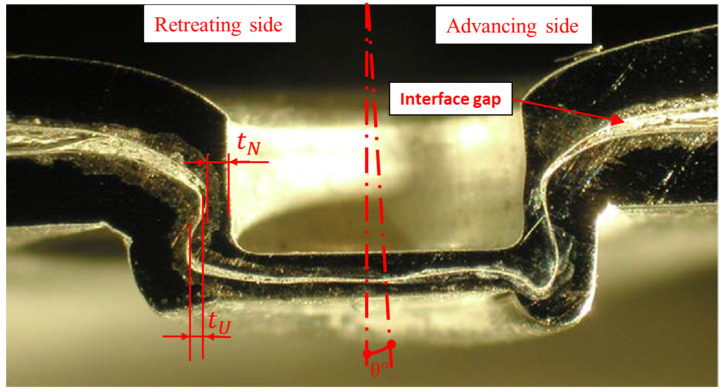
Cross section of a steel clinched joint has a diameter of 6.0 mm on the die side [28].

It is encouraging to qualitatively compare the tool misalignment results in this study with that found by Varis [28] who case studied the production problems of a metal coated steels clinching process in a mass production context and found that tool misalignment could lead to unexpected (additional upward warping and gaps between the sheets) and failed joints (Figure 21).

The notable features of the misaligned case are (with reference to Figure 20 and Figure 21):

- Loss of wall thickness on the advancing side, and gain of wall thickness on the retreating side.
- Loss of interlock on the advancing side, to the point of total collapse thereof.
- Severe thinning compression of the upper sheet at the advancing side. This may be due to stretching by the incoming punch. The bottom sheet does not have noticeably different compression.
- Complete filling of bottom groove at the advancing side and incomplete filling of bottom groove at the retreating side.

**Figure 22:**
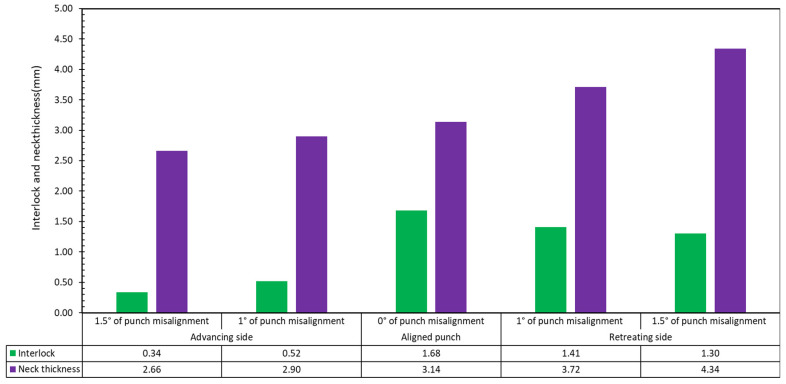
Bar chart of interlock for joint 12–8 mm with 0°, 1° and 1.5° misalignment (S4, S7 and S8).

The interlock decreases as the angular misalignment of the punch increases. The interlock of 0° misalignment is 1.68 mm. The maximum and minimum interlock of 1° misalignment are 1.41 mm and 0.52 mm. The interlock of 1.5° misalignment is the worst (Figure 22). With regard to the neck thickness, the maximum neck thickness of each degree misalignment is higher than that of 0° misalignment. The minimum neck thickness of each degree misalignment is lower than that of 0° misalignment. Tentatively, this implies that, for clinching in steel, assuming a 10:1 scale, that a 1° of punch misalignment could result in a significant reduction in interlock. However, it is possible for misalignment to occur due to the long service life of a machine in the clinching process.

### 3.6. Variability in Interlock

The recorded mean plasticine interlock for 12–8 mm was 1.50 mm, with some variability. From an industrial clinching perspective, a large and constant interlock is desirable. For a metal joint of total 2 mm, the plasticine model suggests an interlock of 0.15 mm, assuming a 10:1 scale.

### 3.7. Overall Discussion

#### 3.7.1. Implications of the Work

Industry practitioners might like to consider placing the thicker sheet on top to produce a stronger joint. In addition, note that misalignment is exceedingly critical, with small values of the order of 1.5 deg causing significant degradation of interlock. Friction between sheets should be enhanced rather than reduced, for better interlock.

The current research identifies that, for a given tool design, there is only an optimum of sheet thickness combination. Using thicker or thinner sheets, or inverting their order, leads to loss of interlock.

Designers of automated assembly lines, e.g., for whiteware, might consider designs that minimize deflection of the support structures. In addition, to take care of specifying tolerances for mounts of punch and die, with a particular need to reduce lateral and angular offsets. Tolerance stack may need to be considered, along with accurately machined geometric alignment features. Designers might also consider the stiffness of the structures if operating multiple punches concurrently.

Angular misalignment increases the risk that the joint may be peeled open from one side preferentially (advancing side). Designers should be cautious about racks of clinched joints that might all be misaligned similarly and oriented unfavorably compared to external loads.

#### 3.7.2. Limitations of the Work

- Even though punch and die were manufactured with an accuracy of 0.05 mm, there are still errors in the punch and die which will affect the results of plasticine.
- Consideration has not been given to the influence of room temperature and indoor humidity on the plasticine material.
- There is a thickness tolerance of each group of plasticine sample, and the mean tolerance range is ±0.1 mm.
- The effect of blank holder was neglected.

#### 3.7.3. Recommendations for Future Studies

This study dealt with establishment of a method for plasticine clinching. The feasibility of the plasticine analogue model in reproducing the behavior of clinching process was explored. Furthermore, the influence of sheet-metal thickness deviation and tool misalignment as potential difficulties in the clinching process in production lines was investigated by using the plasticine multiscale model. There are, however, still many technical issues to be explored. Furthermore, the literature review showed that, in each aspect of this process, there are just a few studies which in time can be expanded to better understand press forming. What follows are just some ideas that could be the focus of future studies:

➢ Establishing the FEA model to evaluate the results of plasticine clinched joints according to this study;
➢ Studying the effect of blank holder force on plasticine analogue model;
➢ Studying the effect of preheating on plasticine analogue model;
➢ Investigating the impact of different lubricants in plasticine analogue model;
➢ Studying the effect of misalignment on tools life using force measurement;
➢ Utilizing a 3D-printed plastic tool for the plasticine analogue.

## 4. Conclusions

With regard to the objectives, this study has confirmed the representativeness of plasticine as an analogue model for steel clinching. In particular, the results are comparable regarding interlock, neck thickness, force profile, and evolution of the strain and its representation in microstructure.

The effect of thickness tolerance, tool misalignment and sheet placement (top vs. bottom) has been investigated. The plasticine results show significant effects for sheet placement are lubricant, thickness of samples and tool misalignment. Compared with glycerine, silicone oil has a lower coefficient of friction, which may be the cause of the reduced interlock. Additionally, the optimal stacking of sheets is a 12–8 sample (12 mm as upper sheet & 8 mm as lower sheet; lubricant is glycerine) with 1.87 mm of interlock among four test samples. Moreover, thickness tolerance showed a critical relationship with interlock, namely a reduction to about half that of the nominal, for both maximum and least material conditions. Furthermore, with an increase of the angular misalignment of punch, the interlock decreases. There is confidence that these findings also apply to steel clinched joints because similar effects are reported in the literature [28,46] and in the steel samples included in the present study.

The plasticine also allows a qualitative understanding of the plastic flow features. The evolution of the force to displacement (stiffness) relationships for steel and plasticine show identical qualitative characteristics. This indicates that the rheology of the process is similar in both mediums.

This work makes a novel contribution of providing a qualitative appreciation of the effects of sheet placement, sheet thickness tolerance, and tool misalignment on the quality of clinched joints. The results have been demonstrated for plasticine analogue modelling, and comparison with clinched steel sheets shows that the cross sections are qualitatively similar. The study provides some confidence that the principles and relationships described may be applicable to other materials. An advantage of the plasticine method is that it allows the intra-sheet behaviour to be visualised. In contrast, this is difficult to do with metallic materials and the smaller scale of commercial clinching.

Plasticine analogue modelling provides a useful method for characterising the deformation involved in a clinch joint. Furthermore, it also reproduces many of the force characteristics:

- plasticine analogue model can be calibrated to a range of hard to soft sheet metals by adjusting temperature and lubricants;
- layers would move on each other if the process time increases as the friction between the layers causes damages at the top layers;
- controlling the thickness tolerance for values under 0.1 mm is so difficult. therefore, using a multiscale model would be very helpful;
- friction has a significant effect on the interlock of clinch joints.

## Figures and Tables

**Table 1:** Different configurations of plasticine slab used in an analogue modelling experiment.

Test No.	Sheet Configuration	Test Purpose	Upper Sheet Thickness [mm]	Lower Sheet Thickness [mm]	Total Thickness [mm]	Total Layers
S1	8–12	Configuration of sheets	8	12	20	20
S2	12–8	Sensitivity to layers quantity	12	8	20	2
S3	12–8	Glycerine vs. silicon oil lubricant	12	8	20	20
S4	12–8	Base model and Thickness tolerance	12	8	20	20
S5	11–7	Thickness tolerance	11	7	18	18
S6	13–9	Thickness tolerance	13	9	22	22
S7	12–8	Angular misalignment 1°	12	8	20	20
S8	12–8	Angular misalignment 1.5°	12	8	20	20

**Table 2:** Interlock of each layer in experiments S4 (12–8) and S1 (8–12).

Layer No.	Interlock (mm)	
	S4 (12–8 mm)	S1 (8–12 mm)
1	0.00	0.00
2	0.30	0.05
3	0.60	0.15
4	0.92	0.20
5	1.14	0.26
6	1.37	0.32
7	1.59	0.51
8	1.68	0.64
9	1.68	0.77
10	1.71	0.81
11	1.71	0.82
12	1.64	0.84
13	1.53	0.75
14	1.39	0.70
15	1.15	0.69
16	0.94	0.69
17	0.74	0.66
18	0.49	0.60
19	0.16	0.40
20	0.00	0.00

**Table 3:** Interlock of each layer in experiments S3 (12–8 mm) with silicone lubricant.

Layer No.	Interlock (mm) S3 (12–8 mm)
1	0.00
2	0.25
3	0.38
4	0.55
5	0.65
6	0.82
7	0.99
8	1.08
9	1.05
10	1.12
11	1.14
12	1.11
13	1.06
14	0.98
15	0.90
16	0.72
17	0.57
18	0.36
19	0.12
20	0.00

**Table 4:** Interlock of each layer in experiments S5 (11–7 mm) and S6 (13–9 mm) with glycerin lubricant. Layers are numbered from the bottom.

Layer No.	Interlock (mm)	
	S5 (11–7 mm)	S6 (13–9 mm)
1	0.00	0.00
2	0.42	0.16
3	0.60	0.24
4	0.71	0.38
5	0.83	0.51
6	0.70	0.54
7	0.61	0.65
8	0.75	0.77
9	0.86	0.80
10	0.88	0.76
11	0.95	0.73
12	0.92	0.72
13	0.88	0.68
14	0.81	0.69
15	0.67	0.64
16	0.45	0.61
17	0.16	0.54
18	0.00	0.51
19	-	0.44
20	-	0.33
21	-	0.20
22	-	0.00

## Data Availability

Not applicable.
